# Unlocking Cardioprotective Potential of Gut Microbiome: Exploring Therapeutic Strategies

**DOI:** 10.4014/jmb.2405.05019

**Published:** 2024-06-25

**Authors:** Jun Qu, Fantao Meng, Zhen Wang, Wenhao Xu

**Affiliations:** 1 Department of Internal Medicine-Cardiovascular, YanTai YuHuangDing Hospital, Yantai, Shandong, P.R. China; 2 Department of Internal Medicine-Cardiovascular, LinYi Central Hospital, LinYi, Shandong, P.R. China

**Keywords:** Microbiome dysbiosis, probiotics, prebiotics, synbiotics, TMAO inhibitors, atherosclerosis

## Abstract

The microbial community inhabiting the human gut resembles a bustling metropolis, wherein beneficial bacteria play pivotal roles in regulating our bodily functions. These microorganisms adeptly break down resilient dietary fibers to fuel our energy, synthesize essential vitamins crucial for our well-being, and maintain the delicate balance of our immune system. Recent research indicates a potential correlation between alterations in the composition and activities of these gut microbes and the development of coronary artery disease (CAD). Consequently, scientists are delving into the intriguing realm of manipulating these gut inhabitants to potentially mitigate disease risks. Various promising strategies have emerged in this endeavor. Studies have evidenced that probiotics can mitigate inflammation and enhance the endothelial health of our blood vessels. Notably, strains such as *Lactobacilli* and *Bifidobacteria* have garnered substantial attention in both laboratory settings and clinical trials. Conversely, prebiotics exhibit anti-inflammatory properties and hold potential in managing conditions like hypertension and hypercholesterolemia. Synbiotics, which synergistically combine probiotics and prebiotics, show promise in regulating glucose metabolism and abnormal lipid profiles. However, uncertainties persist regarding postbiotics, while antibiotics are deemed unsuitable due to their potential adverse effects. On the other hand, TMAO blockers, such as 3,3-dimethyl-1-butanol, demonstrate encouraging outcomes in laboratory experiments owing to their anti-inflammatory and tissue-protective properties. Moreover, fecal transplantation, despite yielding mixed results, warrants further exploration and refinement. In this comprehensive review, we delve into the intricate interplay between the gut microbiota and CAD, shedding light on the multifaceted approaches researchers are employing to leverage this understanding for therapeutic advancements.

## Introduction

The term ‘microbiota’ refers to the community of microorganisms that normally reside within human colon. Although they reside in a relatively much smaller space, they are 10 times more abundant in their number as compared to the total cells of an average adult human body [1]. They are comprised of more than 2000 species with a majority of anaerobic bacteria [2]. The colonization of human gut starts right after the birth, however it gradually expands in diversity until the age of 3 years with a persistent composition that is maintained over years [2-6]. These microorganisms are well tolerated by the host immune cells and their number and special diversity greatly varies from person to person. Such diversity greatly depends on a number of factors such as age, geographical location, diet and host genetic makeup [7-9]. Although a vast majority of these microorganisms live in a commensal relationship with human body, some species develop a symbiotic relationship by producing energy though carbohydrate fermentation, synthesizing vitamins such as vitamin K and B12 [10, 11], short chain fatty acids such as acetate, propionate and butyrate [12], and releasing immune modulating molecules [4, 5, 13]. The butyrate synthesized by the microbiota not only acts as a local anti-inflammatory agent, it also serves as a pivotal energy source of gut epithelium [14, 15]. The symbiotic gut microflora also contribute in digesting the cardioprotective high fiber mediterranean diet [16].

Over the past many decades, our knowledge regarding the health benefits of gut microbiota has been merely limited to vitamin synthesis and immunomodulation. However, the tremendous advancements in molecular biology in recent years have opened new horizons in microbiome research. The number and diversity of gut flora is immensely related to an individual’s overall health and wellbeing. For instance, the microbiome derived short chain fatty acids exhibit strong immunomodulatory [17], analgesic [18], antidepressant [12] and metabolic properties [19]. The emerging scientific evidence suggests a direct link between the composition of microbiome and the pathogenesis of many different diseases. For instance, the neurodegeneration and demyelination in multiple sclerosis has been found associated with dysbiosis in gut microbiome [20]. Likewise, the deformities in the proportion of microbial species are reported to pave the way for hepatic cirrhosis and fibrosis [21], thyroid defects [22], gynecological disorders [23] and inflammatory bowel disease [24]. More importantly, the relevance of microbiota to the cardiovascular system has gained tremendous attention among researchers. Many recent studies have reported a direct relevance of microbiome dysbiosis to the progression of ischemic CADs, atherosclerosis and CAD [25, 26]. Since the composition of gut flora is not constant and is greatly influenced by geographical and diet factors, one cannot directly relate CAD with a particular composition of microbiota. Nevertheless, a deep understanding of different molecular mechanisms that the gut bacteria utilize to modulate cardiovascular function suggests novel therapeutic targets in the prevention and treatment of cardiovascular ailments. An in-depth relationship between CAD pathogenesis and microbiome dysbiosis is briefly summarized in the sections below.

## Pathophysiological Basis of Microbiome Induced CAD

**Fig. 1:**
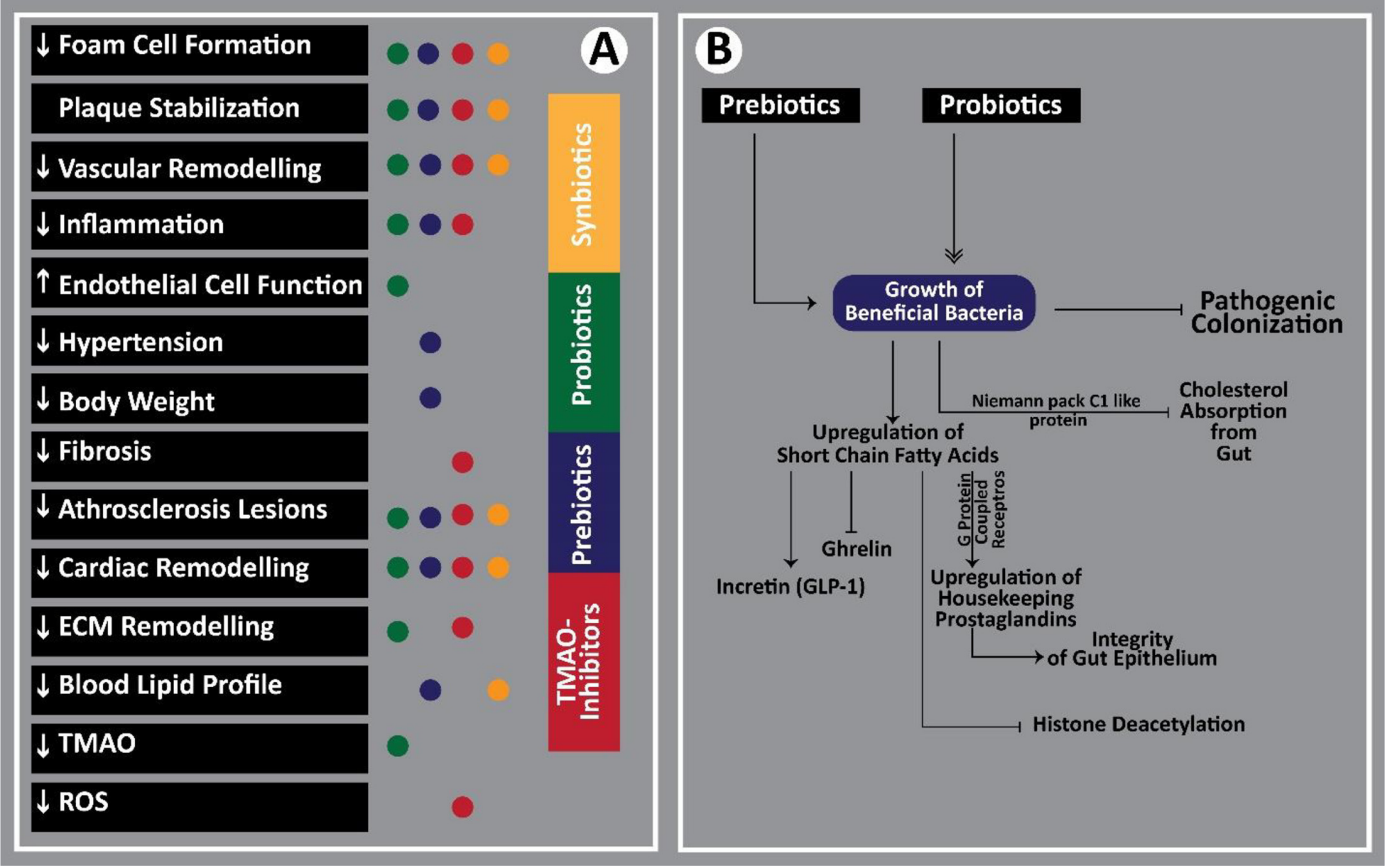
Association between therapeutics interventions to treat microbiome dysbiosis clinical goals. (**A**) illustrates therapeutics effects of probiotics, prebiotics, symbiotics and trimethylamine N-oxide (TMAO) inhibitors. (**B**) depicts various mechanisms employed by probiotics and prebiotics to impart health benefits.

The complicated interrelation between CAD and microbial dysbiosis involves several molecular pathways influenced by a variety of local factors such as infections, host lipid profile, gall-bladder competencies and the regulation of bile secretions, leaky gut with associated endotoxins and microbial metabolites that exacerbate atherosclerosis. For instance, the proinflammatory cytokines are upregulated during infections. The persistently higher levels of these cytokines may compromise the integrity and stability of atherosclerotic plaques. Consequently, these plaques may rupture or decompose and thereby induce clot formation and other complications. Although the relevance of gut infections to the said atherosclerotic complications are not clear [27], the respiratory infections are strongly associated with systemic inflammation and the resultant plaque rupture [28]. The relevance of reverting dysbiosis by administering microbial preparations have been closely associated with several clinical benefits in patients with CAD, as illustrated in Fig. 1. A previous study has reported the presence of bacteria such as *Actinobacteria*, *Firmicutes* and *Bacteroidetes* in the atherosclerotic plaques of Russian population [29], the plaques do not typically contain alive bacteria. Instead, the bacterial lipopolysaccharides and DNA could be detected within the atherosclerotic plaques. Such bacterial remnants are believed to enter the bloodstream vial oral cavity or leaky gut and they may trigger systemic inflammation and atherosclerosis [30]. The role of gut flora in bile acid deconjugation is well documented. A previously reported preclinical study in mice have suggested a microbiome induced dysregulation of cholesterol metabolism via farnesoid X-receptors [31]. Further, the clinical evidence affirms the role of microbiome in modulating bile acid synthesis and thereby affecting gut-liver axis via intestinal and hepatic farnesoid X receptors [32]. The leaky gut is now considered as the most important factor in microbiome induced atherosclerosis. The leakage of bacterial endotoxins such as the Gram-negative lipopolysaccharides (LPS) into the systemic circulation may exacerbate low grade persistent inflammation that may contribute to the development of vascular plaques [33-37]. The host immune cells detect bacterial LPS via special surface receptors such as the toll-like receptors (TLRs). Once activated, these immune cells release proinflammatory cytokines such as interleukin 1 and tumor necrosis factor alpha to induce systemic inflammation.

The lipid metabolism is also affected by the gut flora, however the impact of microbiome dysbiosis on the expression of low density lipoprotein (LDL) is not clear yet [38]. The growing evidence suggests the involvement of peroxisome proliferator-activated receptors (PPAR) in facilitating the crosstalk between host cells and gut flora [39]. Moreover, such crosstalk between the host and microbiota involves interactions that are specific for various microbial species. For instance, the gut flora produce butyrate that further facilitates the β-oxidation process via involving PPARγ receptors. Additionally, PPARγ signaling ensures downregulation of nitric oxide synthesis to maintain anaerobic environment in colon to support the survival of anaerobic microbiota [39-42]. It is evident from the preclinical studies in mice that a high fat diet resulted into significant compositional and spatial modifications in gut microbiota with aberrant PPARγ signaling. Interestingly, the restoration of PPARγ signaling reverted all the compositional and spatial alterations back to normal, suggesting a direct involvement of these receptors in preventing microbiota dysbiosis [43].

In addition to the mechanisms mentioned above, some microbial metabolites are now believed to exhibit direct atherosclerotic properties. One important metabolic dysregulation in microbiome is the aberrant tryptophan metabolism. Under healthy conditions, the gut bacteria metabolize tryptophane to derivatives that upregulate the secretion of GLP-1, an important incretin hormone by activating aryl hydrocarbon receptors. The inability of gut microbiome to metabolize tryptophane is found associated with hyperinsulinemia that has exacerbated metabolic syndrome in both preclinical and clinical studies [44, 45]. Another important bacterial metabolite is the trimethylamine N-oxide (TMAO) that has been reported to be associated with atherosclerosis in rodents [46]. The administration of TMAO via oral route has significantly upregulated serum triglyceride and cholesterol levels in mice [47]. The Higher levels of TMAO are positively associated with the non-alcoholic fatty liver disease and CAD [48-50]. TMAO is a metabolic product of trimethylamine (TMA) which is synthesized by gut bacteria from choline. TMA is metabolized by the hepatic flavin-containing monooxygenases to synthesize TMAO. TMA is produced by many bacterial strains such as *Clostridium XIVa*, *Escherichia coli*, *Escherichia fergusonii*, *Edwardsiella tarda*, *Proteus penneri*, *Anaerococcus hydrogenalis*, *Providencia rettgeri* and *Eubacterium* strain AB3007 [51, 52]. Most of these TMA synthesizing bacterial species are ubiquitously found in mammalian gut [53], hence the involvement of TMA and its metabolite TMAO in CAD pathogenesis could be directly associated with gut microbiome. Many meta-analyses and clinical studies have positively associated upregulated TMAO levels with high cardiovascular risk [54-57]. However, some of these clinical studies have presented conflicting results and more work is required to affirm causality [58-60].

A recent positional paper on coronary pathophysiology and microcirculation by the European Society of Cardiology working Group has outlined various clinical reports of observational nature that have been conducted with pathophysiological rationale [61, 62]. A number of such studies reported microbiome dysbiosis in patients with atherosclerosis and other cardiovascular ailments. Although a few such reports presented conflicting results due to differences in patient background and other technicalities, the alterations in gut microbial diversity has been a prominent pathological factor in most of the patients with atherosclerosis and infarction. Accordingly, *Prevotella* and *Bacteroides* genera were found less abundant than *Escherichia* and *Strptococcus* genera in patients with atherosclerosis [63]. Moreover, species like *Enterobacter aerogenes* and *Klebsiella* were more abundant in such patients as compared to the butyrate producing *Faecalibacterium* cf. *prausnitzii* and *Roseburia intestinalis*. Likewise, the beneficial bacteria such as *Faecalibacterium*, *Bacteroides* and *Prevotella* were less abundant as compared to the opportunistic strains such as *Desulfovibrio*, *Megasphaera*, *Oscillibacter* and *Enterobacter* in patients with high risk of stroke [64]. Interestingly, the metabolic properties of gut flora in diseased subjects were different from those of the healthy subjects, with more pro-inflammatory characteristics in the diseased subjects as compared to the healthy ones [63]. These results were further attested by the findings of another cohort study wherein the proportion of *Enterococcus* was greatly increased in gut flora of the CAD patients. Further, the population of *Eubacterium rectale*, *Roseburia*, *Faecalibacterium* and *Subdoligranulum* were found depleted up to significant levels [65]. Moreover, the metabolic properties of gut flora in CAD patients were also found in agreement with the previously discussed studies. For instance, the gut flora of CAD patients in this large cohort study was enriched with LPS synthesizing bacteria with aberrant tryptophane metabolism.

**Fig. 2:**
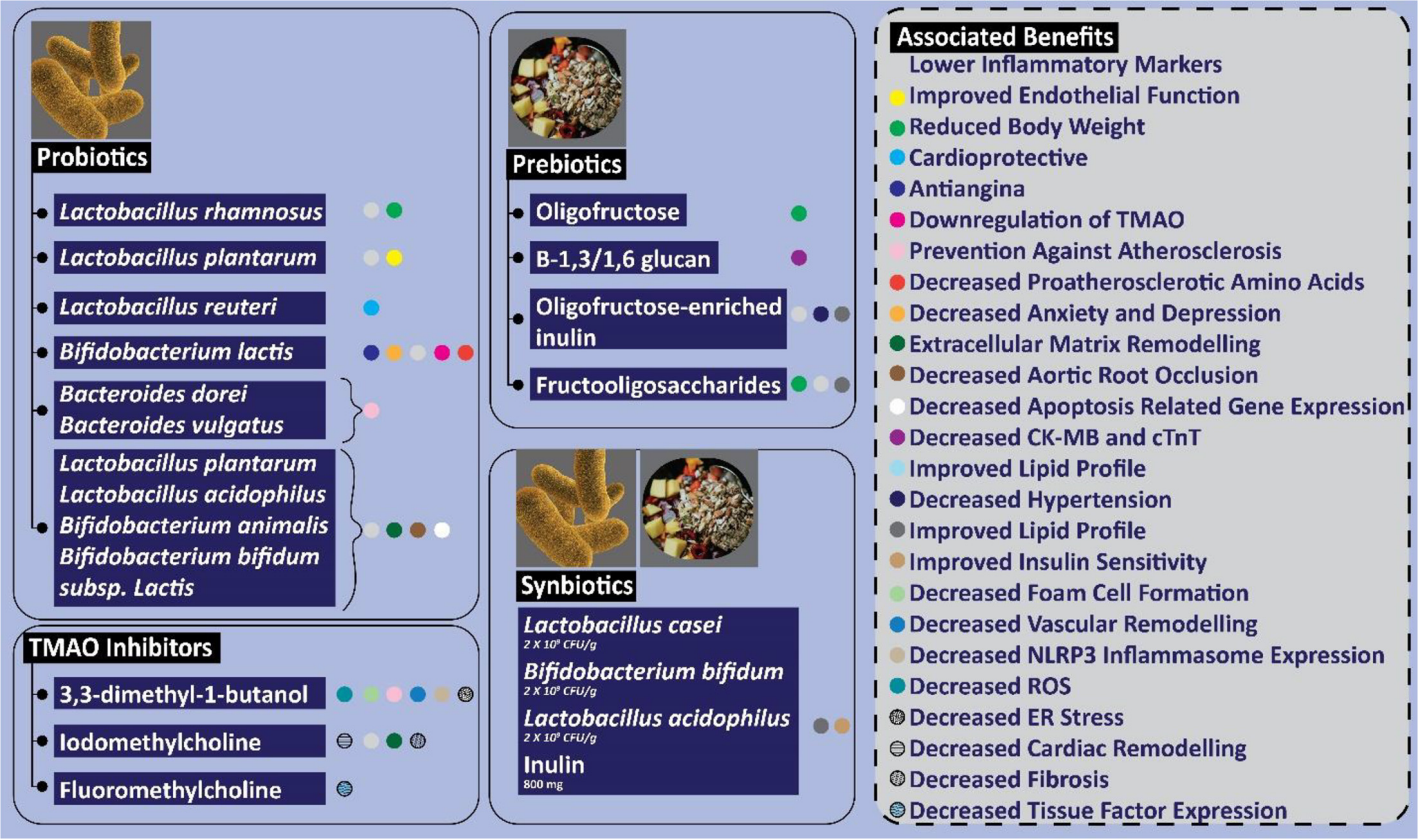
Association between various interventions to revert microbial dysbiosis and clinical goals in treating cardiovascular ailments.

Despite of a strong scientific evidence that links microbiome dysbiosis with CAD pathogenesis, the factors that cause such dysbiosis in CAD are not known till today. Nevertheless, the findings of such studies suggest many valuable therapeutic interventions that could be used as an efficient tool to improve patient’s health and well-being. Some of the valuable associations between such therapeutic interventions and clinical success are illustrated in Fig. 2.

## Contemporary Treatment Approaches

The contemporary interventions employed to inhibit microbiome dysbiosis include prebiotics, probiotics, synbiotics and TMAO inhibitors.

## Prebiotics

These are the substances that are not digested by the gastrointestinal system; however they may be utilized to revert dysbiosis of gut microbiome. By promoting growth and diversity of gut bacteria, these prebiotics may add significant health benefits [66]. For example, chitosan oligosaccharides, pectin polysaccharides, fructooligosaccharides, falactans (galactooligosaccharides), inulin, betaglucan and minolest are prebiotics that are commonly used in patients with cardiovascular disorders. Their health benefits in patients with CAD are well affirmed by clinical trials. Different underlying mechanisms are suggested such as an increase in the growth of beneficial bacteria, improvement of leaky gut by enforcing gut epithelial junctions and the upregulation of short chain fatty acid synthesis in gut bacteria [67]. Prebiotics are reported to reduce the release of ghrelin from gastric mucosa with a significant upregulation in glucagon-like peptide-1 (GLP-1) release [68]. By promoting the growth of beneficial bacteria such as *Bifidobacterium* and *Lactobacilli*, these prebiotics antagonize the colonization of pathogenic species [69]. Likewise, the prebiotic induced synthesis of short chain fatty acids inhibits deacetylation of histone with a profound local anti-inflammatory effect within the gut environment [70-74]. Moreover, the enhanced synthesis of these short chain fatty acids strengthens the integrity of epithelial linkages via G protein receptor 43 [75]. It is important to mention here that the European Food and Safety Authority does not acknowledge the health benefits of prebiotic preparations despite of all clinical and scientific evidence [76].

The benefits of prebiotics in CAD are supported mainly by preclinical studies with only a handful clinical reports [67, 77, 78]. Different prebiotic preparations with potential cardioprotective properties include oligofructose [68], B-1,3/1,6 glucan [79], oligofructose-enriched inulin [80] and fructo-oligosaccharides [81]. The treatment with beta glycans has shown to reduce the risk of ischemia and reperfusion injury after coronary artery bypass [79]. Likewise, the growth of beneficial bacteria such as *Faecalibacterium*, *Lactobacilli*, *Escherichia*, *Alistipes*, *Phascolarctobacteria* and *Lactococci* was significantly increased in CAD patients who received chitosan oligosaccharides [82]. The chitosan preparation also reduced plasma triglycerides and cholesterol levels in CAD patients. The use of prebiotics in metabolic disorders with is well supported by preclinical and clinical studies [83]. For instance, fructooligosaccharides are reported to reduce weight and downregulate pro-inflammatory cytokines in obese rats [81]. Similarly, a randomized controlled trial in human diabetic patients have shown promising improvement in lipid profile with a significant reduction in proinflammatory cytokines after administering inulin and placebo [80]. In another randomized controlled trial in human patients, the administration of oligofructose exhibited marked weight loss with a significant decline in ghrelin release [68]. In two separate randomized controlled trials, inulin [84] and galactooligosaccharides [85] resulted into an increased growth of *Bifidobacteria* in prediabetic obese subjects. Similar results were reported in another randomized controlled trial where the administration of agave fructans induced growth of two important beneficial bacteria i.e., *Lactobacilli* and *Bifidobacteria* in healthy subjects [86]. Accordingly, treatment with guar gum resulted into an increased growth of *Bifidobacteria*, *Roseburia*/*Eubacterium rectale*, *Costridium coccoides*, *Eubacterium hallii* and butyrate synthesizing bacteria in healthy subjects [87].

## Probiotics

The anti-thrombotic, vascular protective, antioxidant and anti-inflammatory effects are probiotics are well acclaimed [88]. Alive *Bifidobacteria* and *Lactobacilli* are commercially available dietary supplements which demonstrate health benefits when administered in optimal quantities [89]. These supplements play a crucial role in mitigating leaky gut syndrome by impeding the hazardous leakage of bacterial lipopolysaccharides (LPS), inhibiting trimethylamine N-oxide (TMAO) formation, and fortifying intercellular linkages within the gut epithelium [78, 88]. Moreover, these preparations promote cholesterol utilization and secretion of bile salts by bolstering deconjugation of bile acids [61]. By boosting cholesterol utilization and exhibiting anti-inflammatory properties, these supplements inhibit plaque formation and thereby produce their anti-atherosclerotic effect [90]. The administration of probiotic preparations containing beneficial microbes such as *Lactobacillus rhamnosus* [91], *Lactobacillus plantarum* [92], *Lactobacillus reuteri* [93], *Bifidobacterium lactis* [94] and preparations containing combination of *Bacteroides vulgatus* and *Bacteroides dorei* [95] have shown significant improvements in CAD. Likewise, the formulation consisting of *Lactobacillus acidophilus*, *Bifidobacterium bifidum*, *Bifidobacterium animalis*, subsp. *Lactis* and *Lactobacillus plantarum* [96] has also shown promising results in inhibiting atherosclerosis in murine model.

The benefits of administering probiotics in cardiovascular diseases are evident from two preclinical studies. In knockout LDLr-/- mice, the administration of *Lactobacillus reuteri* preparations did not reduce cholesterol levels, however the probiotic exhibited strong anti-inflammatory properties with a promising reduction of infarct size in cardiac ischemia [93]. In another similar study, the administration of *Lactobacillus rhamnosus* GR-1 supplements blocked remodeling of myocardium and thereby retained myocardial efficiency rats induced with acute infarction [97]. *Lactobacilli* are considered as the most important prebotic for cardiovascular health. In a preclinical study in swine model, the supplementation of *Lactobacillus plantarum* has exhibited significant antioxidant and anti-inflammatory effects in diseased ischemic model via NF-E2 related factor 2 [98]. The administration of *L. plantarum* in 77 dyslipidemic patients conferred significant synergistic boost in simvastatin induced improvement in the lipid profile and a reduction in calculated cardiovascular risk [99]. The anti-inflammatory properties of *L. plantarum* supplements in CAD patients are further affirmed by other clinical studies as well [92, 100]. However, these supplements did not affect TMAO levels or metabolic parameters in these studies. Likewise, in another double blind clinical trial in 44 CAD patients, the administration of *Lactobacillus rhamnosus* supplements for 12 weeks with caloric restriction exhibited tremendous weight improvement and profound anti-inflammatory effect [91]. These effects were significantly different from those patients who observed caloric restriction without receiving probiotic supplements. Similarly, *Lactobacillus paracasei* (DTA81) has also been reported for its promising antihyperglycemic, anti-inflammatory and lipid lowering properties in patients with atherosclerosis [101]. According to a recently published meta-analysis, *Lactobacillus acidophilus* preparations are the probiotics with strongest lipid lowering potential as compared to the other probiotic preparations [102].

In addition to *Lactobacilli*, the anti-atherosclerotic potential of *Bifidobacteria* is well reported. In a randomized double blind controlled trial, a probiotic Probio-M8 with alive *Bifidobacterium lactis* in combination with atorvastatin and metoprolol was administered to CAD patients [94]. The improvement in anxiety, angina and depression was assessed by Self-Rating Anxiety Scale, Seattle Angina Score and Self-Rating Depression Scale respectively. The findings of this controlled trial exhibited tremendous improvement in anxiety, angina and depression of patients who received probiotics as compared to the control group that did not receive alive microorganisms. Further, the administration of probiotic for 6 months lowered LDL and IL-6 levels up to significant levels as compared to the patients who received placebo.

Participants who received probiotics exhibited a decrease in the abundance of *Parabacteroides johnsonii* and *Flavonifractor plautii*, alongside an increase in *Bifidobacterium adolescentis*, *Bifidobacterium animalis* and *Butyricicoccus porcorum* within their gut microbiota. Noteworthy changes included a reduction in trimethylamine N-oxide (TMAO) and proatherogenic amino acids like l-leucine and l-valine, coupled with an elevation in beneficial microbial metabolites among individuals in the study group. In addition to its well-documented anti-inflammatory and hypolipidemic properties, the probiotic strain may have induced changes in the gut microbiota and metabolome composition, potentially contributing to the observed enhancements in quality of life. However, data on other probiotic strains, such as *Limosilactobacillus fermentum* remain insufficient. In a study involving Wistar rats fed a high-fat diet, administration of a probiotic formulation containing *Limosilactobacillus fermentum* restored gut microbial balance and mitigated metabolic abnormalities, including improvements in blood pressure levels. [103]. The formulation exhibited significant antioxidant and anti-inflammatory effect on myocardium in high-fat-diet female Wister rats [104]. Likewise, in ApoE-deficient mice, the administration of probiotic preparations containing alive *Bacteroides dorei* and *Bacteroides vulgatus* attenuated the formation of atherosclerotic plaques [95].

Preclinical investigations have provided valuable insights into the concurrent administration of multiple probiotics. In a study, probiotics including *Lactobacillus acidophilus*, *Bifidobacterium bifidum*, *Bifidobacterium animalis* subsp. lactis and *Lactobacillus plantarum* were orally administered alone or in combination to male LDLr−/− mice maintained on a far rich diet for 12 weeks. [96]. Mice that received combination of probiotic exhibited a notable reduction in the occlusion of aortic root, coupled with a decline in the lipid content of plaques with signs of plaque stabilization and macrophage infiltration, as well as an upregulation of actin in α-smooth muscle cells. Moreover, the study group displayed a downregulation of multiple genes associated with crucial pathways, including apoptosis, cell adhesion, translocation and metabolism of lipids, remodeling of extracellular matrix and inflammation. Furthermore, the probiotic preparations stimulated the formation of foam cells and promoted proliferation as well as migration of smooth muscle cells, while also reducing macrophage and monocyte proliferation and monocyte migration that was associated with monocyte chemoattractant protein-1 (MCP-1). Likewise, treatment with *Limosilactobacillus fermentum* Y57 and *Lactobacillus rhamnosus* FM9 exhibited promising benefits in upregulating good cholesterol i.e., HDL levels. These results were well-comparable with the effects of a lipid-lowering drug that was administered to the male Wistar rats on high-fat diet [105, 106]. Concurrently, there was a noticeable trend towards a substantial decrease in blood pressure, with systolic blood pressure decreasing by approximately 5 mmHg and diastolic blood pressure by around 2 mmHg. Despite the promising findings from the aforementioned research, it's crucial to note that many probiotic health claims often lack robust support from available data. The optimal probiotic strain(s) and dosage for effectively treating CAD remain unclear.

## Gut Health Strategies: Antibiotics, Postbiotics, Synbiotics

The attempt to target microorganisms within atherosclerotic plaque in CAD using antibiotics has not yielded any observed benefits and was recently discovered to have adverse effects on human health [107, 108]. Furthermore, this approach should be discarded due to its detrimental effects on numerous beneficial gut microorganisms. Analysis of microbial presence within atherosclerotic plaques has revealed a diverse community of over 50 bacterial species, including *Staphylococcus* species, *Proteus vulgaris*, *Klebsiella pneumoniae*, *Chlamydia pneumoniae*, *Chlamydia trachomatis*, and *Streptococcus* species [109]. Most of these bacteria are migrated to the coronary arteries from their natural habitat within oral cavity or gut via bloodstream [109]. An alluring hypothesis has emerged suggesting that the inflammation induced within the plaques by these bacteria may impact the progression of atherosclerosis. One of the preliminary investigations supporting a potential association of CAD with microorganisms found that the cardiovascular events in patients with myocardial infarction were well predicted by the upregulated levels of antibodies against *Chlamydia pneumonia* with a significant abatement in this risk seen upon azithromycin administration [110]. However, these findings were not confirmed by the results of subsequent larger-scale studies that documented no benefits of gatifloxacin or azithromycin in secondary event prevention [111, 112]. A Cochrane meta-analysis analyzed the data of 26000 CAD patients who received quinolones and macrolides. The findings of this meta-analysis suggested that the use of antibiotics did not prevent cardiovascular events in CAD patients. Contrarily, the mortality rate was significantly increased in patients who received antibiotics, suggesting a harmful impact of these antibiotics in CAD [107].

The probiotics are defined as inanimate microbial preparations and/or their components that are administered to exhibit health benefits [113]. The definition however does not affirm postbiotic-associated clinical benefits in CAD patients. Although the purified metabolites of microorganisms with known health benefits do not fall within the definition of “postbiotics”, the fragments of bacterial cell-wall meet such criterion. One of such preparation, Anuc_1100 that is consisted of purified membrane proteins of *Akkermansia muciniphila* have recently shown promising health benefits in diabetic mice [114], suggesting a potential use of such formulations in treating atherosclerosis.cjscjs

Synbiotics comprise a blend of live bacteria and their substrates designed to foster health benefits [115]. They have emerged as promising interventions in human CAD and its associated conditions such as obesity and diabetes mellitus [116]. The favorable effects of synbiotics primarily arise from the synergistic actions of their pre-and probiotic components. A thorough examination of randomized controlled trials [117], has unveiled significant enhancements in various metabolic parameters among patients with metabolic syndrome who underwent synbiotic supplementation. These enhancements encompass reduced levels of serum triglycerides, insulin, LDL cholesterol, total cholesterol, body weight, waist circumference, serum interleukin-6 and blood pressure along with upregulated HDL cholesterol. Nevertheless, determining the optimal synbiotic formulation for CAD treatment remains challenging. In a randomized controlled trial of diabetic patient with cardiovascular comorbidities [118], a synbiotic blend containing inulin, *Lactobacillus casei*, *Lactobacillus acidophilus*, and *Bifidobacterium bifidum* was linked to a promising success in upregulating HDL cholesterol and controlling glycemic levels. Similarly, another trial demonstrated favorable effects on lipid levels among type 2 diabetes patients who received a combination of inulin and *Lactobacillus sporogenes* [119].

Fecal transplantation is a clinical procedure primarily employed in treating *Clostridioides difficile* infection [120]. Nevertheless, researchers have delved into its potential benefits for cardiometabolic health, especially concerning atherosclerosis and potentially CAD. Notably, the transfer of gut microbiota from healthy lean donors to the recipients with marked metabolic syndrome exhibited significant incline in insulin sensitivity. Moreover, the intervention also resulted into an increased colonization of butyrate-producing beneficial bacterial including *Eubacterium hallii* and *Roseburia intestinalis* [121].

Expanding on this observation, a small-scale randomized controlled trial on metabolic syndrome assigned 20 male participants to undergo either autologous fecal or vegan donor transplantation [122]. While a shift towards a gut microbiota profile resembling that of vegan donors was noted in the recipients, this change did not correspond to reductions in vascular inflammation or trimethylamine-N-oxide (TMAO) as indicated by ex vivo production of pro-inflammatory cytokines by the peripheral blood monocytes or evaluated by imaging.

The intricacies of these clinical studies in human patients are complemented by pre-clinical investigations in laboratory animals. For example, the transplantation of fecal bacteria in murine model of myocarditis exhibited profound anti-inflammatory effect by modulating the *Firmicutes*/Bacteroidetes ratio [123]. This discovery holds particular significance considering that an imbalanced gut microbiome skewed towards pro-inflammatory species could potentially contribute to the atherosclerotic initiation and progression [124].

Furthermore, the findings of a recent investigation on atherosclerosis-prone C1q/TNF-related protein 9-knockout mice suggested a strong association between gut microbiota and CAD progression [125] The transplantation of fecal microbiota from wild-type mice transformed the composition of the recipient mice's gut microbiota to impede the progression of atherosclerotic lesions in the carotid artery following partial ligation. These findings underscore the potential of fecal transplantation as a therapeutic approach for mitigating atherosclerosis.

## Modulating TMAO Levels

The scientific interest in trimethylamine-N-oxide (TMAO) has sparked numerous investigations aimed at inhibiting its production and exploring associated outcomes. In an early study conducted by Wang *et al*. [126], researchers discovered that 3,3-dimethyl-1-butanol (DMB) that is choline structural analogue, effectively inhibited TMA-lyase activity. This inhibition led to reduced levels of both TMA and TMAO in mice subjected to L-carnitine or a high-choline diet. Intriguingly, these reductions were accompanied by improvements in cardiac function and decreased foam cell formation in CD1 male mice fed a fat rich diet with no significant effect on body weight or cholesterol levels, as outlined in [127]. Histological examinations revealed anti-fibrotic and anti-inflammatory effects, contributing to the cardioprotective effects of treatment. Similar findings were replicated in a type 2 cardiorenal syndrome murine model [128], where DMB exhibited further antioxidant, anti-inflammatory and vascular protective properties. Moreover, in aged C57BL/6N and Fischer-344 rats, the treatment with DMB elicited similar beneficial effects [129, 130].

In a recent study, DMB was administration with either high-TMAO or high choline diet in wild type murine model of partial carotid artery ligation [131]. Compared to control groups, DMB treatment effectively mitigated adverse vascular remodeling induced by the diets, attenuating flow-induced atherosclerotic lesion formation and suppressing the expression of NLRP3 inflammasome, endoplasmic reticulum stress burden, and reactive oxygen species formation. Roberts *et al*. explored the significance of the microbial TMA-generating enzyme pair, CutC/CutD [132], proposing multiple halomethylcholines capable of preventing platelet activation and thrombus formation in vitro without an associated bleeding risk. Among these molecules, iodomethylcholine and fluoromethylcholine, which inhibit TMA-lyase activity akin to DMB, have been evaluated in subsequent studies [133]. Histological analyses revealed reductions in inflammatory, fibrotic, and extracellular matrix remodeling markers in animals treated with iodomethylcholine, alongside beneficial alterations in cholesterol and bile acid metabolism in wild-type C57BL/6J mice subjected to a high-cholesterol diet [134].

**Fig. 3:**
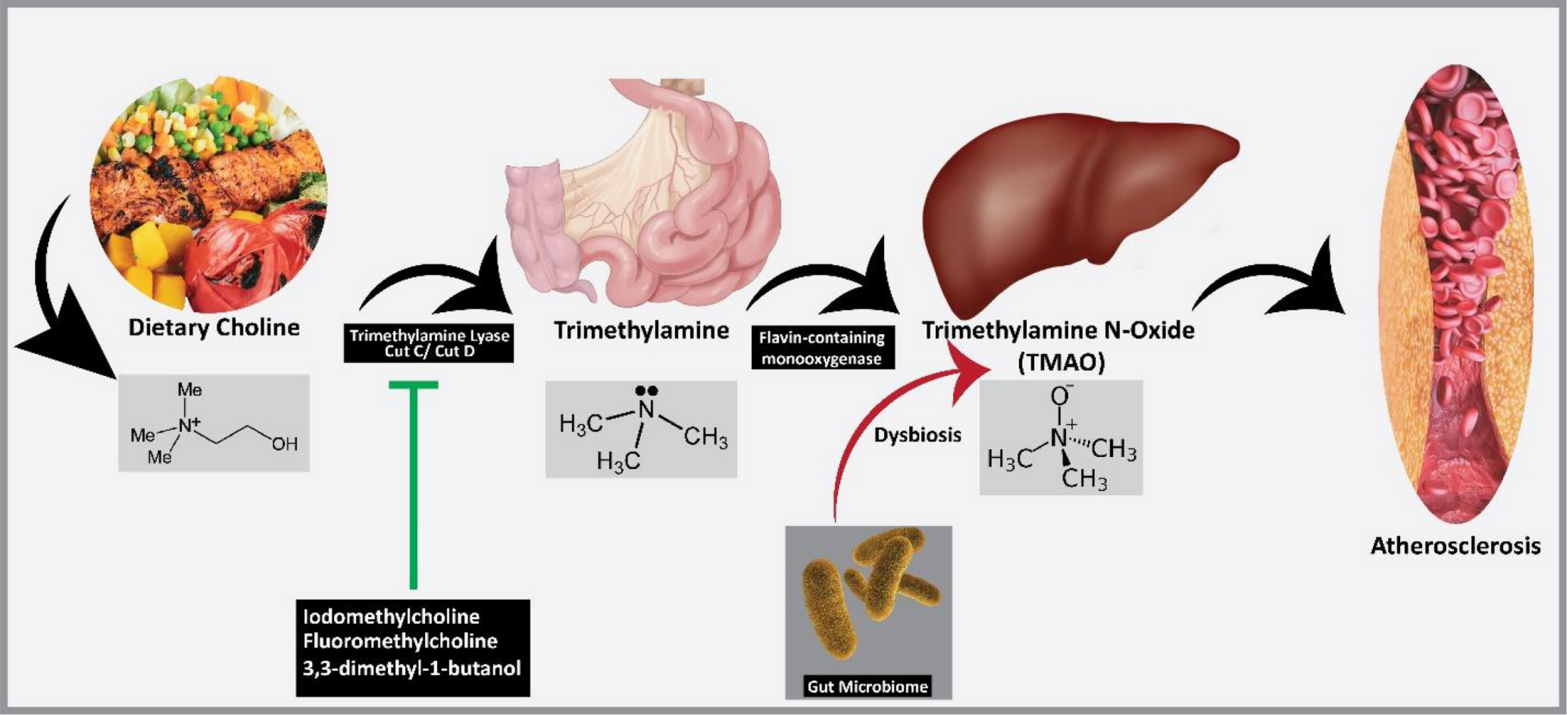
The pathophysiological mechanism of trimethylamine-N-oxide (TMAO) induced atherosclerosis. The dietary choline is converted into trimethylamine within gut via trimethylamine lyase. The trimethylamine is further converted into TMAO by hepatic flavin-containing monooxygenases. The synthesis of TMAO is supported by microbiome dysbiosis. By inhibiting the enzymatic activity of trimethylamine lyase, TMAO inhibitors such as 3,3-dimethyl-1-butnol, fluoromethylcholine and iodomethylcholine may exert potential antiatherosclerosis by attenuating TMAO synthesis.

The pathophysiological mechanism of TMAO induced atherosclerosis is concisely presented in Fig. 3. Fluoromethylcholine demonstrated the ability to reverse TMAO-induced tissue factor expression in a mouse model of arterial injury, suggesting a potential antithrombotic role [135]. In essence, inhibiting TMAO formation may hold promise in attenuating the progression of atherosclerosis by targeting various pathological processes, including foam cell formation, inflammation, endoplasmic reticulum stress, oxidative stress, coagulation, and extracellular matrix remodeling. Flavonoids, a diverse group of polyphenolic compounds found abundantly in various foods such as tea, citrus fruits, berries, red wine, apples, and legumes, have emerged as potential inhibitors of TMAO formation, sparking interest in their role in cardiovascular health. Among these compounds, flavonoid aglycones such as baicalein, fisetin, acacetin, and myricetin have demonstrated significant binding affinity to TMA-lyase, the enzyme involved in TMAO production. Similarly, flavonoid glycosides like baicalin, naringin, and hesperidin also show promising inhibitory effects on TMA-lyase activity [136]. Thus, targeting TMA-lyase activity presents a novel approach for reducing TMAO levels, potentially contributing to the observed benefits of flavonoids in preventing CAD [137]. However, the association between dietary patterns, such as the Mediterranean diet, and TMAO levels remains under debate, with conflicting findings and potential sex-specific associations observed [138, 139]. Consequently, further investigation is necessary to clarify the role of specific dietary patterns in modulating TMAO levels.

In conclusion, while initial research suggests the potential of flavonoids and dietary patterns in modulating TMAO and preventing CAD, evidence in humans remains limited. Therefore, the use of TMAO inhibitors or other methods to manipulate gut microbial composition for CAD prevention purposes is currently under investigation. It is premature to recommend routine microbiota measurements or modulation strategies for this specific purpose without further substantiation through rigorous research.

## Conclusion

Currently, a substantial body of evidence links disruptions in gut microbial balance, known as dysbiosis, to CAD and associated cardiovascular risks. This connection is mainly attributed to the induction of mild systemic inflammation involving activities or components of gut bacteria, representing a series of fundamental pathophysiological events. Moreover, the metabolites that are generated by the gut microbiota act as indicators of heightened cardiovascular risk among patients, although definitive causality in human populations has yet to be firmly established. While several therapeutic strategies aimed at modulating the microbiota have been proposed and tested, their effects have generally been modest. Despite some relatively robust evidence derived from small-scale randomized controlled trials and meta-analyses, the translation of microbiota manipulation into clinical practice for CAD management will likely remain a distant prospect until larger, well-designed randomized controlled trials are conducted and their outcomes assessed.
